# Depauperate Avifauna in Plantations Compared to Forests and Exurban Areas

**DOI:** 10.1371/journal.pone.0000063

**Published:** 2006-12-20

**Authors:** David G. Haskell, Jonathan P. Evans, Neil W. Pelkey

**Affiliations:** 1 Department of Biology, University of the South Sewanee, Tennessee, United States of America; 2 Environmental Science and Studies, and Information Technology, Juniata College Huntingdon, Pennsylvania, United States of America; University of St. Andrews, United Kingdom

## Abstract

Native forests are shrinking worldwide, causing a loss of biological diversity. Our ability to prioritize forest conservation actions is hampered by a lack of information about the relative impacts of different types of forest loss on biodiversity. In particular, we lack rigorous comparisons of the effects of clearing forests for tree plantations and for human settlements, two leading causes of deforestation worldwide. We compared avian diversity in forests, plantations and exurban areas on the Cumberland Plateau, USA, an area of global importance for biodiversity. By combining field surveys with digital habitat databases, and then analyzing diversity at multiple scales, we found that plantations had lower diversity and fewer conservation priority species than did other habitats. Exurban areas had higher diversity than did native forests, but native forests outscored exurban areas for some measures of conservation priority. Overall therefore, pine plantations had impoverished avian communities relative to both native forests and to exurban areas. Thus, reports on the status of forests give misleading signals about biological diversity when they include plantations in their estimates of forest cover but exclude forested areas in which humans live. Likewise, forest conservation programs should downgrade incentives for plantations and should include settled areas within their purview.

## Introduction

Native forests are shrinking worldwide. Human settlement, clearing for agriculture, and conversion of forests to tree plantations account for most of these declines [1]. This loss of natural systems has resulted in declines in biodiversity and degradation of ecosystem services formerly provided by the forests [2]. In response to these declines, many governments and private agencies have put into place forest conservation and management initiatives [3], [4]. Because these initiatives usually occur in an environment of limited financial and political resources, the relative impacts of different land use changes has to be understood in order to assess and prioritize the merits of different potential conservation schemes [5]. Yet, comparisons of the effects of different types of forest loss are seldom made. The literature on forest loss and biodiversity is dominated by investigations of the effects of single types of forest loss. For example, the effects of timber extraction on biodiversity are well understood for many regions [6], [7]. Likewise, the responses of biodiversity to different types of agriculture [8], [9] or urbanization [10], [11] have been elucidated for some areas of the world. But few studies have compared the effects of timber extraction, human settlement or agriculture within one landscape using a standardized methodology that allows statistically rigorous assessments of the relative impacts of different types of forest loss. Because forests are usually subject to losses from multiple sources, such broad-level comparisons are a prerequisite to informed conservation planning.

Despite the paucity of rigorous comparative data, commentators in the scientific literature and in the news media have drawn conclusions about the relative impacts of different types of forest loss. These conclusions mostly rank human settlements as more damaging than forestry activities [10], [12], [13]. Hansen et al.'s [11] review of exurbanization and biodiversity highlights this assumption in the previous literature, concluding that the “general view among conservationists and the public is that exurban development alters ecological processes and biodiversity to a greater extent than forestry and agriculture”. Yet, the few available data paint a more complex picture wherein some intensively managed timberlands may impact biodiversity as much as, or more than, human settlements [14]. In general, intensively urbanized landscapes have much profound effects on native biological diversity than do lightly settled areas [10], [11]. Therefore, the relative impacts of forestry and urbanization will likely depend on the intensity of urbanization and the type of forest management, but these comparisons have yet to be made for most areas of the world [14], [15], making any conclusions about the relative impacts of forestry and exubanization premature.

We used the avifauna in forests on the Cumberland Plateau in the southeastern United States as a case study. We use a consistent sampling and analysis protocol to compare the effects of different types of forest loss on avian biodiversity. The forests of the southeastern United States, including the Cumberland Plateau, are an excellent region in which to perform such a case study because their loss of native forest to tree plantations and human settlements conforms to the global pattern of loss of native forest cover [12], [16], [17]. All native forest types are in decline in the southern United States (1% per year in the area in which this study was conducted [16]) and this decline is predicted to continue for the foreseeable future [12]. On the Cumberland Plateau, housing development and conversion to pine plantations are the primary causes of these reductions [16], [17]. The Cumberland Plateau and the Southern Appalachian region in which the Plateau sits harbor very high levels of biological diversity. These areas have been identified as areas of very high conservation priority–one of the “Global 200” biodiversity hotspots [18], [19]. Our objectives therefore were: (1) to quantify avian diversity in the main habitat classes in our study region, (2) to perform a statistically rigorous comparison of diversity across habitat classes, and (3) to assess and compare the conservation value of each habitat class across the landscape.

## Results

Bird surveys (n = 503) were located in one of six distinct habitat classes (described in more detail in the Methods section): mature native hardwood forests (n = 85 points, 9 transects), thinned native forests (n = 30 points, 3 transects), mature loblolly pine (*Pinus taeda*) plantations (n = 54 points, 6 transects), mid-aged loblolly pine plantations (n = 75 points, 8 transects), early loblolly pine plantations (n = 69 points, 7 transects), and exurban areas (n = 190 points, 19 transect). 82 species were detected (Table S1).

**Figure 1:**
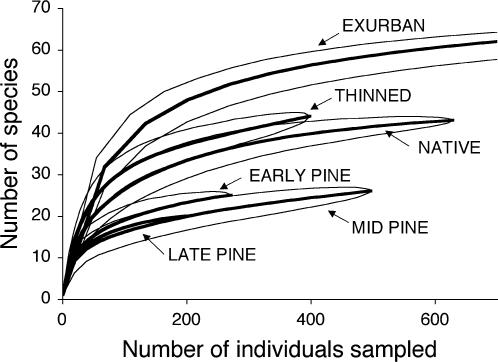
Species richness in each habitat class. Richness is shown by rarefaction curves which describe how the number of species changes with the number of individuals sampled, thus controlling for both sampling effort and bird density. Thick lines indicate mean richness and thin lines indicate 95% confidence intervals. Note that the “late pine” line hugs the first part of the “mid pine” line, ending at two hundred individuals and twenty species.

The six habitat classes differed significantly in species richness. At the scales of habitat classes (Figure 1) and transects (Figure S1), exurban areas had the highest richness, followed by thinned native forests, then native forests, then all age-classes of pine plantation. A similar pattern emerged at the level of individual counts, except that native forests did not differ from mid-aged pine plantations in the number of species detected per count (Figure S2, Table S2).

**Figure 2:**
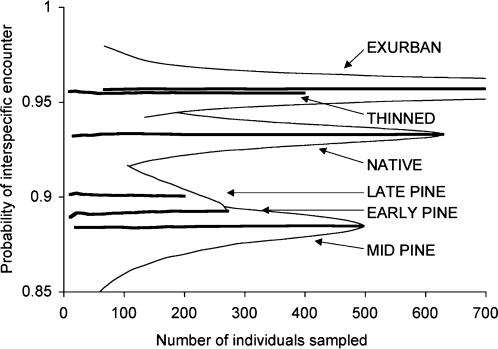
Species evenness in each habitat class. Evenness is shown by the probability of interspecific encounter (PIE, shown by thick lines; thin lines show 95% confidence intervals). PIE controls for both sampling effort and bird density, and uses repeated re-sampling of the data to calculate the probability that the next bird sampled will be of a different species. Therefore, high PIE values indicate high species evenness. See “caveats” section of Discussion for an analysis of how detectability differences might influence the curves in this figure.

Evenness measured at the scale of habitat classes was also highest in exurban areas, followed by thinned native forests, then native forests, then all age-classes of pine plantation (Figure 2). At the smaller scale of transects, the same pattern emerged, except thinned forests had slightly higher evenness than exurban areas (Figure S3).

**Figure 3:**
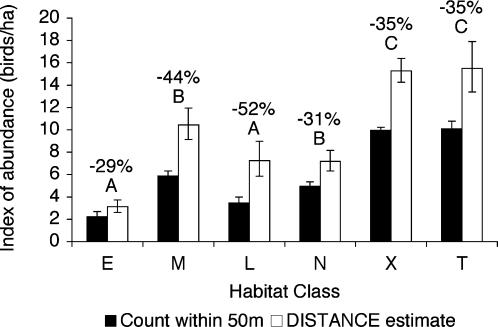
Indices of bird abundance for six habitat classes. Filled bars show means and standard errors of indices of abundance calculated by counting all birds within 50 m of each count center. Habitat classes with the same letter are not significantly different from one another in a Tukey HSD multiple means comparison calculated using this data. Open bars show estimated abundance with 95% confidence intervals from DISTANCE software using all birds detected up to 150 m. Numbers above bars show the percentage difference between the DISTANCE estimate and the estimate made by counting birds within 50 m of count centers. (E = early pine plantation, M = mid-aged pine plantation, L = late pine plantation, N = native forest, X = Exurban areas, T = thinned native forest.)

The methods used to estimate indices of abundance gave somewhat different estimates. DISTANCE software [20] consistently estimated higher abundances than the method of counting birds within a 50 m radius (Figure 3; Table S2). Counting birds within 50 m of count centers gave proportionally lower estimates for mid-aged and late pine plantations than it did for other habitat classes (Figure 3). Both methods gave similar ranking of abundance, however. Indices of abundance were highest in exurban and thinned areas, followed by native forests, mid-aged, and late plantations, followed by early plantations (Figure 3).

**Figure 4:**
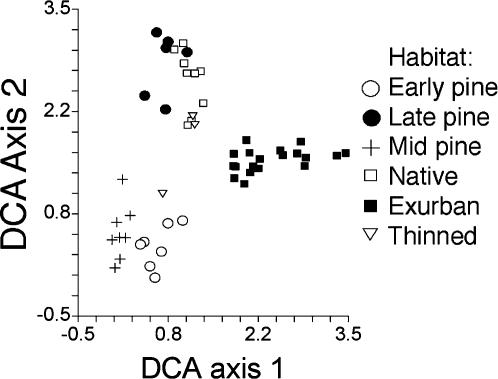
Detrended correspondence analysis of bird communities calculated at the scale of transects. Each point represents the position in ordination space of the bird community detected at one transect. The two axes show the relative position of each transect in the multi-dimensional space defined by the species found on each transect. Thus transects with similar bird communities cluster together on the graph. The first axis (DCA 1) is the one along which most of the variation in the ordination space is arranged (eigenvalue = 0.56), the second axis (DCA 2) is the second most important axis through the ordination space (eigenvalue = 0.44).

The detrended correspondence analysis (DCA) ordination showed that the bird communities were distinct in most habitat classes, especially when these communities were examined at the level of transects (Figure 4), but these patterns were also apparent at the level of individual points (Figure S4). Exurban areas clustered away from all other classes. Early and mid-aged plantations clustered next to each other away from all other classes. Native forests and late plantations overlapped each other in ordination space and thinned native forests overlapped some native forests, or sat in the center of the ordination space. The extent of variation along the first DCA axis (a measure of beta diversity) also differed significantly among habitat classes (squared deviation from mean MRPP standardized test statistic = −21.5, p<0.001; absolute deviation from median MRPP standardized test statistic = −26.0, p<0.001). Exurban areas had higher variability among points and transects than did the other habitat classes.

**Figure 5:**
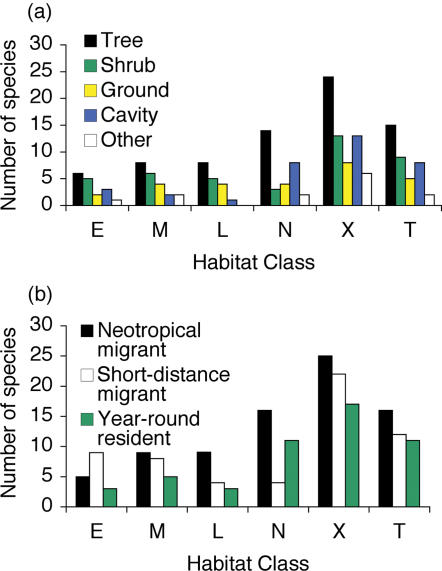
Life history characteristics of birds in each habitat class. (a) Numbers of species nesting in different nest site types in each habitat class, (b) Numbers of species with different migratory patterns in each habitat class. (E = early pine plantation, M = mid-aged pine plantation, L = late pine plantation, N = native forest, X = Exurban areas, T = thinned native forest.)

The richness of species with different life history characteristics differed among habitat classes. Pine plantations of all age classes had lower richness of cavity- and tree-nesting species, neotropical migrants, and year-round residents than did all other habitat classes (Figure 5; Nest sites: ^2^ = 73.5, df = 20, p<0.05; Migratory status: χ^2^ = 58.9, df = 10; p<0.05). Native forests were not significantly different from exurban areas for either type of life history characteristic (Nest sites: χ^2^ = 2.21, df = 4, p>0.05; Migratory status: χ^2^ = 4.84, df = 2; p>0.05).

The habitat classes differed in their conservation value as measured by Partners in Flight (PIF) scores [21], [22]. When 2001 PIF scores were weighted by an index of abundance, exurban areas had the highest scores, followed by thinned and native forests, followed by all ages of pine plantation (MRPP standardized test statistic = −12.17, p<0.001). The same result was obtained with unweighted PIF scores (MRPP standardized test statistic = −24.33, p<0.001). When species were categorized according to 2001 PIF priority ranks and their presence and absence was tallied across habitat classes, the same ranking emerged (Table 1). The continent-wide 2004 PIF data classified according to Watch List or Additional Stewardship species showed the same pattern (Table 1). When habitat classes were compared for the number of PIF species reaching their highest index of abundance in each habitat, native forests had the highest number of species, followed by thinned forests, exurban areas and mid-aged plantations (Table 1).

**Table 1:**
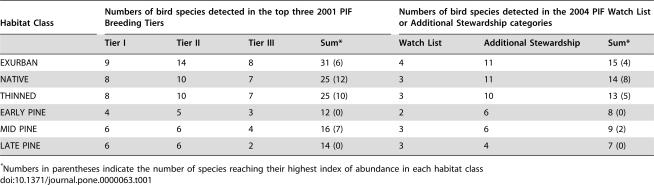
Numbers of Partners in Flight (PIF) species detected in each habitat class.

Habitat Class	Numbers of bird species detected in the top three 2001 PIF Breeding Tiers				Numbers of bird species detected in the 2004 PIF Watch List or Additional Stewardship categories		
	Tier I	Tier II	Tier III	Sum*	Watch List	Additional Stewardship	Sum*
EXURBAN	9	14	8	31 (6)	4	11	15 (4)
NATIVE	8	10	7	25 (12)	3	11	14 (8)
THINNED	8	10	7	25 (10)	3	10	13 (5)
EARLY PINE	4	5	3	12 (0)	2	6	8 (0)
MID PINE	6	6	4	16 (7)	3	6	9 (2)
LATE PINE	6	6	2	14 (0)	3	4	7 (0)

* Numbers in parentheses indicate the number of species reaching their highest index of abundance in each habitat class

**Figure 6:**
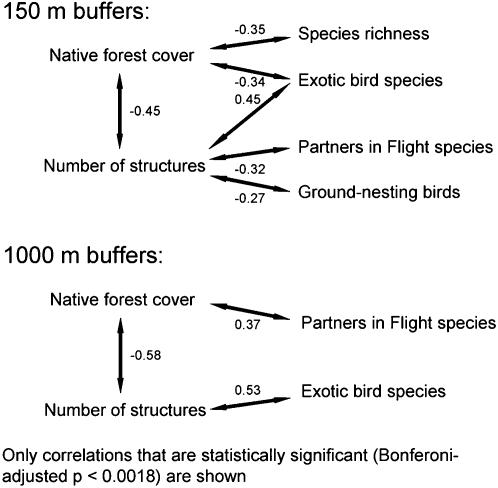
Summary of Pearson correlations between landscape metrics and bird community characteristics. Only statistically significant correlations are shown (see Table 3 for listing of all correlations, regardless of significance). All buffers were calculated around the locations of point counts in exurban areas.

The relationship between landscape structure and bird species richness depended strongly on the spatial scale at which the landscape measure were made (Table 2). For 150 m buffers around transects, species richness increased with edge density, area-weighted mean shape index (AWMSI), and area-weighted mean patch fractal dimension (AWMPFD). These trends were reversed for 1000 m buffers. Within the exurban habitat class, the proportion of native forest was negatively correlated with the number of structures at both the 150 m and 1000 m scales (Table 3; Figure 6). At both scales, areas with more structures had more exotic bird species. At the 1000 m scale, the abundance of PIF priority species increased with forest cover. At the 150 m scale the abundance of ground-nesters and PIF priority species was negatively correlated with the number of structures. Species richness at this scale decreased with native forest cover. Cowbird and avian nest predator abundance were not associated with either housing density or the proportion of native forest at either scale.

**Table 2:**
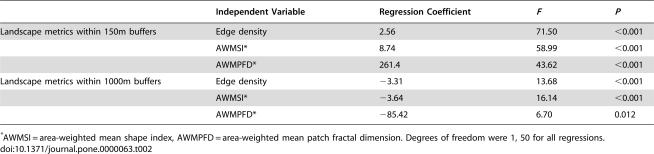
Summary of regression statistics for analyses of the effects of landscape structure and composition on the total species richness of birds detected on transects.

	Independent Variable	Regression Coefficient	*F*	*P*
Landscape metrics within 150m buffers	Edge density	2.56	71.50	<0.001
	AWMSI*	8.74	58.99	<0.001
	AWMPFD*	261.4	43.62	<0.001
Landscape metrics within 1000m buffers	Edge density	−3.31	13.68	<0.001
	AWMSI*	−3.64	16.14	<0.001
	AWMPFD*	−85.42	6.70	0.012

* AWMSI = area-weighted mean shape index, AWMPFD = area-weighted mean patch fractal dimension. Degrees of freedom were 1, 50 for all regressions.

**Table 3:**
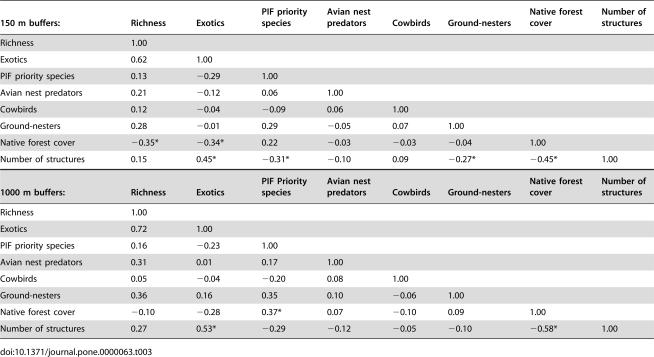
Summary of Pearson correlations among landscape metrics and bird community characteristics within the exurban habitat class. Correlations that are statistically significant (Bonferoni-adjusted p<0.0018) are marked with an asterisk.

150 m buffers:	Richness	Exotics	PIF priority species	Avian nest predators	Cowbirds	Ground-nesters	Native forest cover	Number of structures
Richness	1.00							
Exotics	0.62	1.00						
PIF priority species	0.13	−0.29	1.00					
Avian nest predators	0.21	−0.12	0.06	1.00				
Cowbirds	0.12	−0.04	−0.09	0.06	1.00			
Ground-nesters	0.28	−0.01	0.29	−0.05	0.07	1.00		
Native forest cover	−0.35*	−0.34*	0.22	−0.03	−0.03	−0.04	1.00	
Number of structures	0.15	0.45*	−0.31*	−0.10	0.09	−0.27*	−0.45*	1.00

1000 m buffers:	Richness	Exotics	PIF Priority species	Avian nest predators	Cowbirds	Ground-nesters	Native forest cover	Number of structures
Richness	1.00							
Exotics	0.72	1.00						
PIF priority species	0.16	−0.23	1.00					
Avian nest predators	0.31	0.01	0.17	1.00				
Cowbirds	0.05	−0.04	−0.20	0.08	1.00			
Ground-nesters	0.36	0.16	0.35	0.10	−0.06	1.00		
Native forest cover	−0.10	−0.28	0.37*	0.07	−0.10	0.09	1.00	
Number of structures	0.27	0.53*	−0.29	−0.12	−0.05	−0.10	−0.58*	1.00

## Discussion

### Bird communities and forest change

Biological diversity and conservation status are notoriously difficult to quantify [23]. Therefore, to guard against methodological bias we dissected the data on bird communities with a variety of tools, examining diversity across multiple spatial scales and controlling for sampling effort. We assessed the conservation status of habitat classes by breaking down Partners in Flight data (a measure of the conservation value of species) both quantitatively and qualitatively. A clear signal emerges from these analyses. For all measures of diversity and conservation status, pine plantations were significantly impoverished relative to native forests, exurban areas, and thinned forests. In addition, exurban areas and thinned forests had higher diversity than did native forests. Some measures of conservation status ranked exurban areas and thinned forests above native forests, but other measures ranked native forests ahead of all other habitat classes.

More specifically, all ages of pine plantations had lower species richness than did oak-hickory forests (as reported in previous preliminary analyses [17]). In late (i.e., older) plantations those species that were still present were similar to those found in oak-hickory forests, whereas early and mid-aged plantations were dominated by early successional specialists. This result was not an artifact of dividing plantations into different age classes in our analyses – species richness is low whether or not categories are combined. Pine plantations also had lower evenness than did oak-hickory forests, meaning that the bird communities in plantations were dominated by a few species, rather than having many species of more even abundance. Plantations had either similar beta diversity (early and late plantations) or slightly lower beta diversity (mid-aged plantations) than did oak-hickory forests. Indices of bird abundance in plantations were either lower than native forests (for late and early plantations) or the same (for mid-aged plantations). Plantations had fewer cavity- and tree-nesting birds, and a proportional loss of neotropical migrant birds relative to oak-hickory forests (especially for early and mid rotation plantations). The finding that plantations in our study area supported lower species diversity than did native forests echoes the results of previous studies in both tropical and temperate regions [24]–[26].

The intensive mechanical and chemical preparation techniques used to clear land for pine plantations on the Cumberland Plateau involves the removal of all or most of the vegetation. The plantation is then stocked with one species of tree, although some other species resprout to form a sparse understory. These processes reduce the structural complexity of the plantation and remove most cavity trees from the area. Thus, many cavity-nesting birds are lost and the simplified vertical and horizontal structure of the forest may translate into lower bird diversity. This interpretation is consistent with previous work on the relationship between the structural complexity of habitat and bird diversity [27], [28]. The early stages of pine plantations provide habitat for some early-successional specialist birds such as Prairie Warbler, *Dendroica discolor*, and Yellow-breasted Chat, *Icteria virens*. These species were also found in thinned forests and exurban areas.

The bird community found in exurban areas had higher species richness than that found in oak-hickory forests. Evenness, beta diversity, and indices of abundance were also higher in exurban areas. There were no pronounced differences in the breakdown of nest site usage or migratory types between the bird community in exurban areas and the community in oak-hickory forests, although there was a trend for exurban areas to have proportionally more short-distance migrants and year-round residents (this trend was not statistically significant). Exurban areas provided habitat for both early successional species (e.g., Common Yellowthroat, *Geothlypis trichas*, Chipping Sparrow, *Spizella passerina*, Indigo Bunting, *Passerina caerulea*) and for late successional species (e.g., Pileated Woodpecker, *Dryocopus pileatus*, Wood Thrush, *Hylocichla mustelina*). In addition, a number of species (e.g., Barn Swallow, *Hirundo rustica,* Cedar Waxwing, *Bombycilla cedrorum*, Red-winged Blackbird, *Agelaius phoeniceus*, Purple Martin, *Progne subis*) were found only in areas with human housing and not in other habitat classes. This co-habitation of both forest-dwelling and disturbance-adapted species accounts for the high overall diversity of this habitat class. Previous studies have found that high structural diversity in exurban areas (e.g., mix of forest, ornamental shrubbery, lawns, and urban areas) may promote higher bird diversity [29]. The bird community in exurban areas also, however, included exotic species that are generally considered to have low or negative conservation value (European Starling, *Sturnus vulgaris*, House Sparrow, *Passer domesticus*, Rock Pigeon, *Columba livia*). In addition, although exurban areas supported many forest-adapted species, the abundance of many of these species were lower than in native forests.

Within the exurban habitat class, exotic species increased with the density of houses and other structures, as would be expected from previous research [30]. Ground-nesting birds and PIF priority species also declined with the density of structures (when measured at a small spatial scale, but not at the larger scale). However, neither cowbird or avian nest predator abundance were associated with our measures of urbanization. Forest cover at a large spatial scale was positively associated with PIF priority species, but at a smaller scale forest cover was negatively associated with species richness. Therefore, intensification of exurban settlements had a complex effect on bird diversity, locally increasing richness but decreasing conservation priority species in areas where settlement had removed much of the forest cover. Our study was conducted in a region still dominated by forest, and the exurban areas in our study (using Marzluff et al.'s definitions of “exurban” [31]) did not encompass any heavily urbanized areas of the sort that previous studies have reported as depauperate in avian diversity [30]. Thus, the analyses reported here indicate that the effects of housing development on avian diversity appear to depend heavily on landscape context.

Exurban areas appear to cover a significant portion of the landscape worldwide. For example, exurban areas are the fastest growing landcover type in the United States and they cover more than fifteen times the area of densely settled urban areas [32]. They account for 25% of total area of privately-owned land in the U.S. and 46% of the privately-owned land in the eastern temperate forests in which this study was conducted [32]. As yet, there is no global data available on the area of land that consists of low density human settlement mixed within forests [33]. United Nations data on the extent of so-called “trees outside forests” provides an indirect approach to this question. “Trees outside forests” are defined as trees growing in areas that also support human habitation or agriculture [33]. In Costa Rica, for example, these trees account for over half of the nation's wood production [34]. Likewise, in the State of Kerala, India, 83 percent of the timber volume extracted in the 1990s came from “trees outside forests” or “homesteads” and only 7 percent from “forest areas” [35]. These data suggest that low density residential developments form a substantial portion both of overall land area and of the total area of forest, underscoring the need to understand how biodiversity responds to exurban and rural development.

Thinned forests had higher richness, evenness, and abundance of birds than did unthinned oak-hickory forests. The bird community was a mix of early successional species (e.g., Yellow-breasted Chat, *Icteria virens*) and forest-nesting species (e.g., woodpeckers). The contrast between the high bird diversity found in thinned forests and the low diversity of plantations is striking because in both cases the land is managed for timber extraction. Our data therefore corroborate previous research which suggests that the effects of timber management on biodiversity strongly depend on the site preparation and logging techniques used [36]. In our study, thinned forests were subject to no chemical or mechanical site preparation, and some live and dead trees were retained in the logged area, resulting in a highly diverse bird community. Pine plantations were both herbicided and bulldozed and had little or no tree retention, resulting in low abundance of all but a few bird species.

The comparisons of species richness, evenness, and abundance described above provide one approach to understanding the conservation status of habitat types. Another approach takes a continent-wide perspective by using the priority scores independently developed by Partners in Flight (PIF). These scores are assigned to bird species based on both objective criteria (e.g., data on changes in abundance through time) and subjective criteria (e.g., assessments made by PIF personnel of perceived threats to habitat) [21], [22]. Species with high scores are considered to have higher conservation priority than those with low scores. When these scores were used to compare the bird communities assessed in this study, exurban areas obtained the highest scores, followed by thinned forests, oak-hickory forests, then pine plantations. This finding was the same regardless of which version of PIF data were used or whether the PIF data were weighted by indices of abundance. In addition, when the data were summarized using only birds in high-ranking PIF tiers, the same conclusions emerged. However, native forests ranked higher than all other habitat classes if we based the species tally on the numbers of birds reaching their highest indices of abundance in each habitat class. Because this last method used indices of abundance, not just presence or absence, the method may give a better approximation of habitat quality than do the other methods. Overall, therefore, analyses of PIF data rank pine plantations lowest, but different analyses give different answers about the relative ranks of native forests, exurban areas and thinned forests.

Changes in land cover affect bird populations not only by changing habitat classes (as described above), but by changing the spatial configuration of land covers (e.g., by changing levels of fragmentation). In this study, the direction of landscape effects on breeding bird richness depended on the spatial scale at which we calculated landscape metrics [37]. Increases in edge density, area-weighted mean shape index, and area-weighted mean patch fractal dimension were all associated with increases in species richness when the metrics were calculated within 150m buffers of the transects. This pattern was reversed within 1000 m buffers. Previous studies have reached differing conclusions about the relative effects of large and small scale landscape attributes on bird communities [38], [39]. Our data suggest that on the Cumberland Plateau birds are affected by landscape configurations at multiple spatial scales, with large-scale landscape disturbance decreasing species richness and smaller-scale fragmentation increasing species richness.

### Caveats

This study focused on birds that are visible or audible from morning point counts. Thus, they omit birds that are active at night (e.g., nightjars and owls), or that are inconspicuous at all times (some raptors). The study also likely under-samples birds that sing in the early spring before the return of most migrants (e.g., Brown Thrasher, *Toxostoma rufum*). The study provides no data about the habitat uses of migratory birds, post-breeding birds in late summer, or birds in the winter. The contributions of landscape change to these other aspects of bird community dynamics in this region await investigation.

The data gathered in this study document the presence and diversity of birds, not their breeding success. At one level this does not introduce much bias: birds that are not present in a habitat cannot breed. At another level, studies of abundance can be misleading. For example, we found several forest-nesting species singing from narrow (1–15 m) buffers around streams in clear-cuts. Whether these buffers offer the same quality nesting habitat as unfragmented forest is unknown. Previous studies have found that edge-dominated forest fragments provide sub-optimal nesting habitat for forest-dwelling birds [40]. If this applies to buffers on the Cumberland Plateau, point count surveys that encompass these very narrow strips of forest may be biased towards overestimating the quality of the habitat for forest-dwelling birds. Habitats may also differ in the abundance of nest predators, parasites, and food. In particular, small patches of forest are associated with lower food supply [41] and higher nest predation, although the strength of the edge-related predation effect is variable [42]–[44]. Exurban areas may also have higher densities of exotic predators such as cats. A study on the Cumberland Plateau found that cats were only found at high densities in the centers of urban areas, not in the more sparsely populated exurban and rural areas [45].

Bird detectability may vary across habitat classes, potentially confounding comparisons made by point counts. If birds are harder to detect in some habitats than in others (e.g., because of differences in the density of vegetation), then among-habitat differences in bird abundance may be obscured or magnified, depending on the direction of the detectability bias. In this study the difference between indices of abundance estimated using DISTANCE software [20] and indices of abundance estimated by counting birds within 50 m of count centers gives an indication of the extent of differences in detectability. DISTANCE estimates are based on detectability functions fitted to the data, whereas counts made within fixed areas do not take account of detectability. Therefore, DISTANCE estimates will be less biased by variations in detectability than will counts made within a fixed radius. We found that native forests, exurban areas and thinned forests were all very similar in the percentage difference between DISTANCE estimates and counts within 50 m of count centers (Figure 3). Early pine plantations had slightly smaller differences, whereas late and mid-aged plantations had larger differences. This implies that birds were slightly more detectable in early plantations and were less detectable in late and mid-aged plantations. These observations make biological sense: visibility is very high in early plantations, but is reduced in dense older plantations.

Differences in detectability among habitat classes have the potential to affect the interpretation of some of our analyses, but not others. Our comparisons of richness and evenness are based on analysis methods which control for the number of individuals counted (i.e., the curves measure richness and evenness independent of sampling effort or density). Therefore, our measures of richness and evenness are likely not biased by differences in detectability. One possible exception to the lack of detectability bias in these analyses would occur if the extent of interspecific differences in detectability varied across habitats (e.g., if the proportional detectability differences between species changed across habitats). If this were the case, then the evenness calculation might under-estimate evenness in habitats in which some species are proportionally less detectable than they are in other habitats. Two factors suggest that this is not a substantial bias. First, the initial rate of species accumulation was similar across habitats (Figure 1) and, second, our species rarefaction curves approached saturation (Figure 1), suggesting that detectability biases were not driving the differences in evenness in our study.

The ordination and life history analyses describe which species are present in each habitat and their interpretation is not muddied by differences in detectability, particularly because rarefaction curves (Figure 1) indicate that our species sampling was approaching saturation in all habitat classes. Likewise, most of our analyses of PIF conservation priority data do not use estimates of abundance and are thus not affected by detectability differences. However, two analyses do make use of an abundance index that is derived from count data affected by detectability differences: the weighted analysis of 2001 PIF scores and the categorization of 2001/2004 PIF species by the habitat in which we detected each species at its highest abundance index. These analyses therefore slightly overestimate the conservation value of early pine plantations and underestimate the value of mid-aged and late plantations. The differences in detectability we measured are not large enough, however, to change our interpretation of these data. If all abundance indices are re-weighted by the detectability differences observed in this study (i.e., by the percentage difference between DISTANCE estimates and estimates from simple counts in Figure 3), then the 2001 PIF scores still show that exurban areas had the highest scores, followed by thinned and native forests, followed by all ages of pine plantation (MRPP standardized test statistic = −9.17, p<0.001). Re-weighting abundance indices slightly changes the categorization of 2001 and 2004 PIF data by species reaching their highest abundance index in each habitat (numbers in parentheses in Table 1). Specifically, in the 2001 data one species is moved from the “native” class to “late plantation” and one species from “thinned” to “mid-aged plantation”, and in the 2004 data one species is moved from “thinned” to “late plantation”. These minor changes have no effect on the relative ranking of the habitat classes.

### Implications for forest monitoring and policy

These findings have important implications for the monitoring and conservation of biodiversity. Many global and regional reports on the status of forests include plantations in their estimates of forest cover, but exclude forested areas in which humans live. For example, the Food and Agriculture Organization of the United Nations includes tree plantations in its estimates of total forest cover, but excludes any forested area that contains significant human residential or agricultural use, even when such forested areas account for the majority of both the forest area and the timber supply in many regions [1]. In the United States, the federal assessment of southern forests also includes plantations in estimates of forest cover, but defines any area with “residential” use as non-forest [12]. Thus, forestland converted to plantations is reported as “no loss of forest”, whereas adding houses to a forest is reported as a “loss of forest”. Yet, our data show that plantations have much lower levels of biodiversity than do native forests and that exurban areas can retain much of the biodiversity of native forests. Therefore, current methods of accounting for forests give potentially misleading results for biodiversity analyses. At best, this complicates attempts to quantify the status of forest biodiversity; at worst, it can lead commentators who analyze only summary statistics to conclude that concern about forest change is misplaced [46]. Therefore, we suggest that forest accounting reports separate plantations from forests in their summary statistics, not just within the details of the reports. These summary statistics should also include data on forested areas that contain human settlements. Some regions have started to include areas with human settlements in their forest accounting or have broken out plantations from forests [47], [48]. We recommend that the forest accounting methods of other regions and of the U.N. should follow this lead. We do not mean to imply that plantations have no value for biodiversity or that exurban areas have no harmful effects on biodiversity. Rather, we recommend that forest accounting reports correct their current strong bias towards plantations and away from human settlements, thereby allowing for a more refined and accurate assessment of the status of forests and the biological diversity they contain.

The biological poverty of plantations and the relative richness of some exurban areas also generates significant policy implications. Government tax incentive programs for private land and management guidelines for public land often treat plantations in the same way as forests, opening the public purse for both. Yet, the purse snaps shut if residential development is involved [49], [50]. Many of these policies are in part designed to address conservation of biological resources. Our data suggest that these goals are being undermined by the conflation of plantations with forests and of exurban areas with lost forests. If these policies are to provide benefits for biological diversity, we suggest that they should first downgrade incentives for plantations. Second, forest conservation policies might also benefit from including exurban areas under their purvues. Such broader policies may have the added benefit of enhancing the welfare of humans living within and around forests, rather than treating human residential use of land as “separate” from and in competition with forest conservation. The need for this integration has been identified as one of the key challenges for conservation in the next few decades [51], [52].

## Methods

### Bird Surveys

The composition of bird communities was quantified using 5 minute point counts arranged in transects of 8–10 points. To eliminate among-observer bias all counts were conducted by one observer (DGH). A pedometer was used to space counts within each transect at least 200 m apart. The pedometer was calibrated before conducting the transects. All counts were conducted within 4 hours of sunrise. At each count all birds seen or heard were recorded. The distance between the observer and each bird was estimated using a rangefinder for calibration. Counts were not conducted if the wind speed was above 3 on the Beaufort scale or if it was raining or foggy. Each transect was contained within just one habitat class (defined below) and counts were conducted at least 50 m from the edge of each habitat class (usually more than 150 m).

Each transect was located in one of six distinct habitat classes: mature native hardwood forests (NATIVE), thinned native forests (THINNED), mature loblolly pine (*Pinus taeda*) plantation (LATE), mid-aged loblolly pine plantation (MID), early loblolly pine plantation (EARLY), and exurban areas (EXURBAN). NATIVE sites were dominated by mature trees and showed no evidence of recent logging. They had canopies dominated by oaks (*Quercus* sp.) and hickories (*Carya* sp.), with some Virginia pine (*Pinus virginiana*), shortleaf pine (*Pinus echinata*), and red maple (*Acer rubrum*). These forests had understories of immature trees, blueberries (*Vaccinium* sp.), greenbriar (*Smilax rotundifolia*), and sassafras (*Sassafras albidum*). THINNED sites had between 50% and 90% of the canopy removed but had not been subject to burning, herbicides, or bulldozing. All had been thinned within two years of the bird counts. LATE plantations had completely closed canopies of loblolly pine and had a sparse understory of sassafras, maple, and blueberry. MID plantations had loblolly trees between 0.5 m and 2 m high and had not formed a closed canopy. Grasses, forbs, and *Rubus* bushes grew densely between the pines. Trees on EARLY plantations were shorter than 0.5 m and were separated by ground that had been bared by a combination of one or more site preparation techniques (burning, herbicides, and bulldozing). Other than pine seedlings, they had either no visible living plants or sparse growth of ragweed (*Ambrosia artemisiifolia*) and grasses. EXURBAN sites encompassed areas ranging from suburban (e.g., strip malls, housing developments), through exurban, to rural (farmhouses scattered in a mix of pasture and woodland). Mean housing density within the EXURBAN class fell within Marzluff et al.'s standardized [31] definition of “exurban” (in our study: mean = 3.88 houses per hectare, SE = 0.31; median = 2). There were no significant differences among habitat classes for the dates on which counts were made (Kruskal-Wallace ANOVA, Chi-squared = 10.4, p = 0.11). Counts were made between May 21 and June 19 in 2000 and 2001 and all analyses were conducted on data pooled between years. To check for any strong year-to-year variation, we randomly selected three transects from 2000 for repeat sampling in 2001 and found no significant differences in richness or abundance (single factor ANOVAs for both richness and abundance in one transect from each of NATIVE, EARLY, MID classes; p>0.50, df = 1,19 for all ANOVAs; if data are pooled among all three transects then analyzed with a paired t-test to compare both richness and abundance for the same individual points in both years, p = 0.41, df = 29, t = −0.83 for abundance; p = 0.91, df = 29, t = 0.12 for richness).

### Data analysis

We quantified species richness at three spatial scales: at the level of each point count, at the level of each transect (pooling all points within each transect), and at the level of each habitat class (pooling all transects within each class). For the two larger scales we compared richness using rarefaction curves. These curves describe species richness while controlling for the confounding effect of sampling effort and bird density [53]. We constructed rarefaction curves using EcoSim [54] with 1000 iterations and independent sampling. For the analysis at the level of habitat classes we used the default abundance levels for rarefaction curve construction (S+3 abundance levels up to a maximum of 42, where S = number of species in sample). For the analysis of transects we constructed rarefaction curves for each transect, then calculated the mean and standard error of all transects within each habitat class. Because transects differed in the number of individuals detected, we truncated each transect's rarefaction curve at the abundance level that allowed comparison across all transects within a habitat class–this was the minimum number of individuals recorded on any transect in each habitat class. Thus, we truncated the analysis at 50 individuals for EXURBAN and THINNED, 36 individuals for NATIVE, 30 individuals for EARLY and MID, and 24 individuals for LATE.

For the analysis at the level of individual points there were not enough observations per point to construct meaningful rarefaction curves, so we calculated the number of species at each point and performed a nested ANOVA for richness (transect nested within habitat class; ANOVA calculated using MGLM in Systat (Systat Software Inc., Richmond, California, USA, version 5.2.1)). This point-level analysis therefore controls for sampling effort, but not density (samples with more individuals will likely have more species detections).

We used EcoSim to calculate Hurlbert's [55] probability of interspecific encounter for each transect and for data pooled within each habitat class. This measure of evenness controls for variation in the number of individuals sampled. We used the same EcoSim settings and datasets as the richness analyses.

We used two methods to check the robustness of our conclusions to variation in the technique used to calculate indices of relative abundance. First, we calculated per-point indices of abundance by dividing the number of birds detected within 50 m of each point by 0.79 ha (the area of the 50 m circle). We performed a nested ANOVA on these indices (transect nested within habitat class; ANOVA calculated using MGLM in Systat). Second, we used DISTANCE [20] to calculate indices of abundance using the shapes of detection functions (estimates of how the probability of detecting a bird changes with distance from the point). We used the analytical approach described in Buckland et al. [56] and used Chi-squared goodness of fit, Akaike's Information Criterion values, and visual inspection of detection functions to select models that provided the best fit to the data. We used these models to estimate indices of abundance and 95% confidence intervals for each habitat type.

We estimated beta diversity by conducting a detrended correspondence analysis (DCA) in MVSP [57]. This analysis is an ordination technique that uses reciprocal averaging of species abundance data to place samples (e.g., transects or points) in an ordination space defined by a small number of dimensions. The detrended analysis places samples in the ordination space such that distances between points are equivalent across the entire ordination space, allowing beta diversity to be measured and compared in units of standard deviations of species turnover. We conducted two DCAs: one using all point counts as the sampling units and another using transects as the sampling units (with point counts pooled within each). To assess among-habitat differences in beta diversity we quantified variation along the first axis of the DCA in two ways. In the first, we calculated for each sample (either a point or a transect) the absolute value of the deviation from the median value for the habitat class. In the second, we calculated the square of the deviation from the mean value for the habitat class. Habitat classes with high beta diversity should have large deviations from the mean or median. Because the resulting values could not be transformed to meet the assumptions of parametric tests we used multiresponse permutation procedures (MRPP) in Blossom [58] which make no assumptions about the distribution of the data to assess differences among habitat classes in these measures of beta diversity. Because beta diversity measures variation among sites, we excluded thinned native forests from these analyses due to the small sample size (n = 3) of transects.

We used Birds of North America species accounts [59] and descriptions of nest sites in the literature to code each species by nest site and migratory status. We used Chi-squared analyses to test for differences among habitat classes.

To put our results into a regional and global context we produced indexes using 2001 Partners in Flight (PIF) priority scores for the Cumberland Plateau [21] for all species and habitats in our samples. First, we calculated the number of individuals of each species detected within 50 m of count centers per transect for all habitat classes (an index of relative abundance), then multiplied this by priority scores derived from PIF. This procedure weights all PIF scores by an index of relative abundance. Second, we examined PIF scores unweighted by any measure of abundance. Because the resulting data could not be transformed to meet the assumptions of parametric tests we used MRPP to assess differences among habitat classes. We also categorized species according to PIF ranks and quantified the numbers of species from each habitat class present in each of these PIF priority classes. We also used the 2004 PIF continent-wide categorization [22] and quantified the numbers of “Watch List” and “Additional Stewardship” species from each habitat class. Last, we tallied the numbers of PIF species (using both 2001 and 2004 classifications) reaching their highest index of abundance in each habitat class.

We used a landcover database from the year 2000 to calculate landscape metrics associated with each bird-sampling transect [16]. This database was constructed by digitizing landcovers from aerial photography and satellite imagery, followed by ground-truthing (landcover categories: native forest with >70% canopy cover, thinned native forest with 30–70% canopy, cleared land with mineral soil exposed, pine plantation with >70% canopy cover, pine plantation in preparation, “other landcover” with no canopy, “other landcover” with partial canopy; the “other” categories included pasture, urban areas, and mines). We buffered each transect at 150 m and 1000 m in ArcView (ESRI, Redlands, California) and computed landscape metrics using Patch Analyst [60]. We then used linear regression to compare each landscape metric to bird species richness measured at the level of the whole transect. To more closely examine the effects of urbanization, we quantified the density of structures (houses, barns, commercial buildings) and the proportion of native forest within 150 m and 1000 m buffers around each point in the EXURBAN habitat. These measures and the measures of bird diversity at each point could not be transformed to meet the assumptions of parametric tests. We therefore used Pearson correlations to associate the density of structures and the proportion of native forest cover with species richness, abundance of exotic birds (house sparrows, European starlings, and rock pigeons), abundance of high conservation priority birds (2001 PIF ranks 1 and 2), abundance of cowbirds, abundance of avian nest predators (blue jays, American crows), and abundance of ground-nesters. All measures of bird abundance were made using the number of birds detected within 50 m of the point count center. In cases where the buffers of adjacent points overlapped, only one of the points was used in the analysis.

## Supporting Information

Figure S1. Species richness in each habitat class, calculated at the scale of transects. Richness is shown by rarefaction curves which describe how the number of species changes with the number of individuals sampled, thus controlling for both sampling effort and bird density. Thick lines indicate means of rarefaction curves calculated for each transect and thin lines indicate 95% confidence intervals.
(0.12 MB TIF)

Figure S2. Richness measured at the scale of individual points in six habitat classes. Means and SE are presented. Habitat classes with the same letter are not significantly different from one another in a Tukey HSD multiple means comparison. (E  =  early pine plantation, M  =  mid-aged pine plantation, L  =  late pine plantation, N  =  native forest, X  =  Exurban areas, T  =  thinned native forest.)
(0.10 MB TIF)

Figure S3. Species evenness in each habitat class, calculated at the scale of transects. Evenness is shown by the probability of interspecific encounter (PIE). Thick lines indicate means of rarefaction curves calculated for each transect and thin lines indicate 95% confidence intervals. PIE controls for both sampling effort and bird density, and uses repeated re-sampling of the data to calculate the probability that the next bird sampled will be of a different species. Therefore, high PIE values indicate high species evenness.
(0.17 MB TIF)

Figure S4. Detrended correspondence analysis of bird communities calculated at the scale of individual points. Each point represents the position in ordination space of the bird community detected at one point count. The two axes show the relative position of each point count in the multi-dimensional space defined by the species found at each point count. Thus point counts with similar bird communities cluster together on the graph. The first axis (DCA 1) is the one along which most of the variation in the ordination space is arranged (eigenvalue  =  0.62), the second axis (DCA 2) is the second most important axis through the ordination space (eigenvalue  =  0.52).
(0.51 MB TIF)

Table S1. Indices of abundance for each habitat class. Two numbers are listed for each species in each habitat. The first is the proportion of points in that habitat in which the species was detected. The second is the number of birds of each species detected within 50 m per point for each habitat class divided by the number of counts and the area of the 50 m radius circle. Note that these indices are affected by differential detectability in each habitat and true density will therefore differ from these indices (see Caveats section of the Discussion for further details on detectability). * indicates species detected while traveling between point counts, but not detected during any point counts. These species were not included in statistical analyses.
(0.23 MB DOC)

Table S2. Results of nested ANOVA on the number of species Results are presented for birds detected within 50m of the count center and for all birds detected regardless of distance from count center. Note that these indices are affected by differential detectability in each habitat and true density will therefore differ from these indices (see Caveats section of the Discussion for further details on detectability).
(0.04 MB DOC)
