# Supplementary figures and images for: Depauperate Avifauna in Plantations Compared to Forests and Exurban Areas
Source: PLoS One. 2006 Dec 20;1(1):e63. doi: 10.1371/journal.pone.0000063 (PMC1762314; doi:10.1371/journal.pone.0000063)

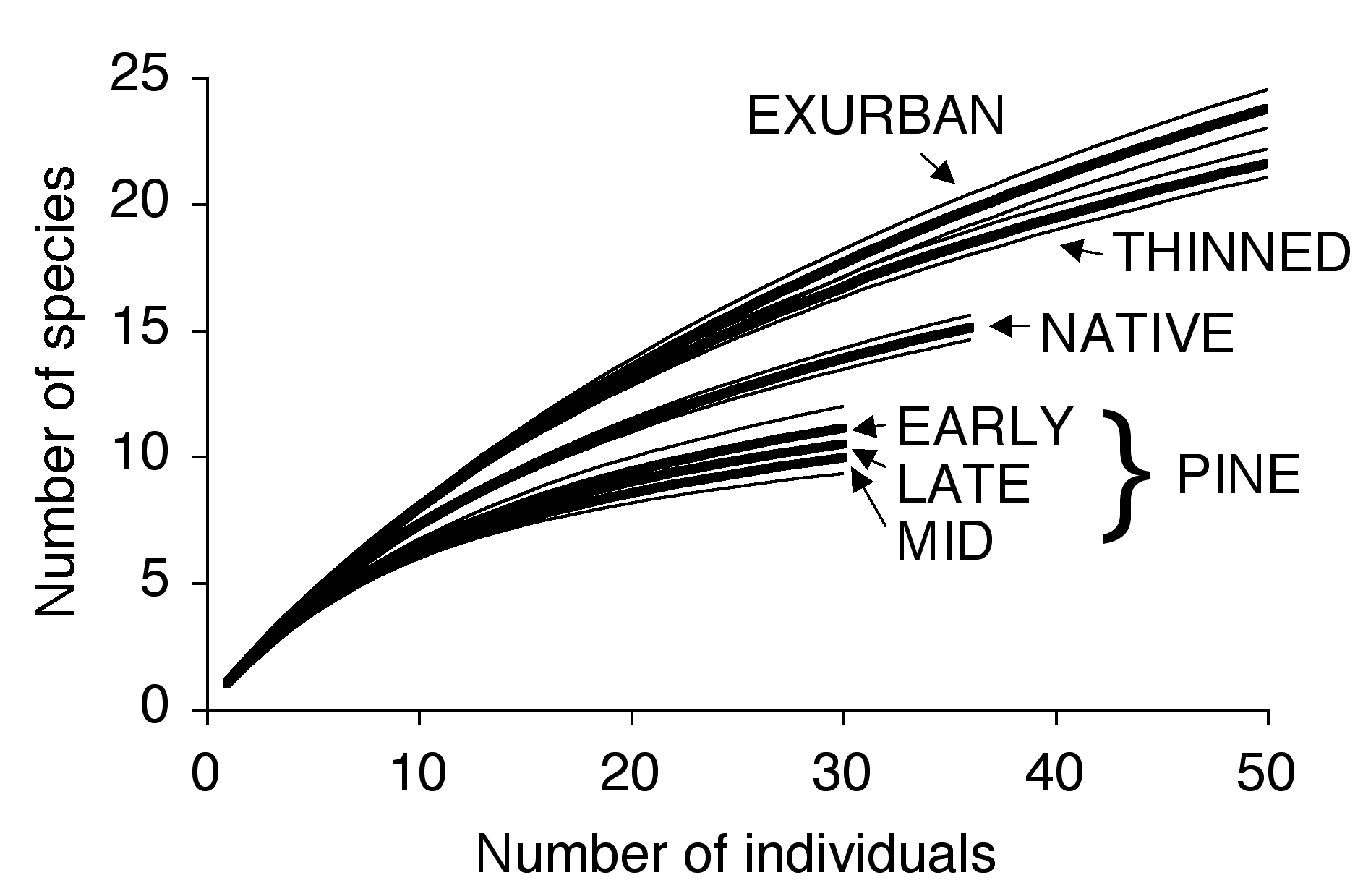

Supplement: Figure S1. Species richness in each habitat class, calculated at the scale of transects. — Richness is shown by rarefaction curves which describe how the number of species changes with the number of individuals sampled, thus controlling for both sampling effort and bird density. Thick lines indicate means of rarefaction curves calculated for each transect and thin lines indicate 95% confidence intervals. (0.12 MB TIF) [file pone.0000063.s001.tif]

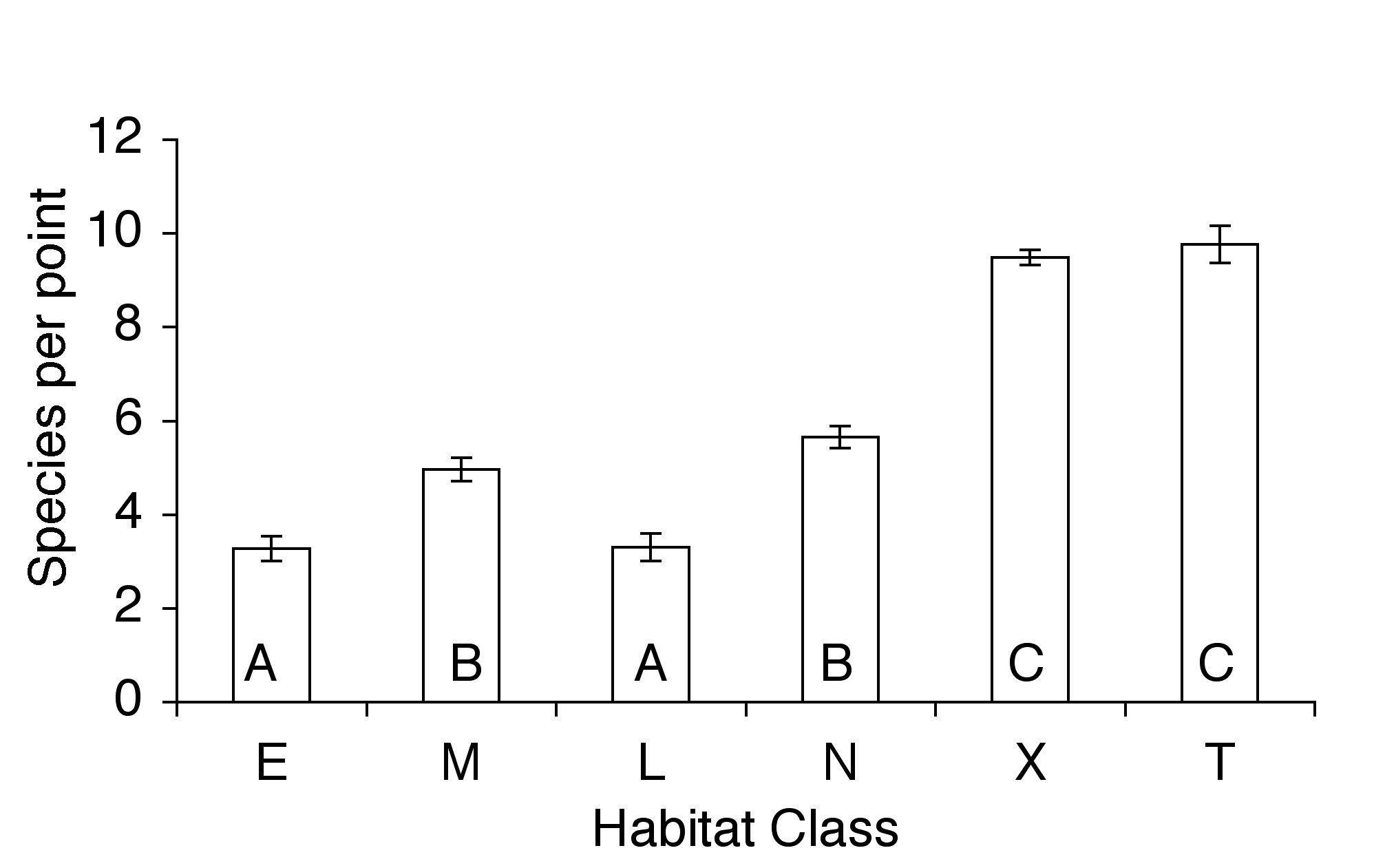

Supplement: Figure S2. Richness measured at the scale of individual points in six habitat classes. — Means and SE are presented. Habitat classes with the same letter are not significantly different from one another in a Tukey HSD multiple means comparison. (E = early pine plantation, M = mid-aged pine plantation, L = late pine plantation, N = native forest, X = Exurban areas, T = thinned native forest.) (0.10 MB TIF) [file pone.0000063.s002.tif]

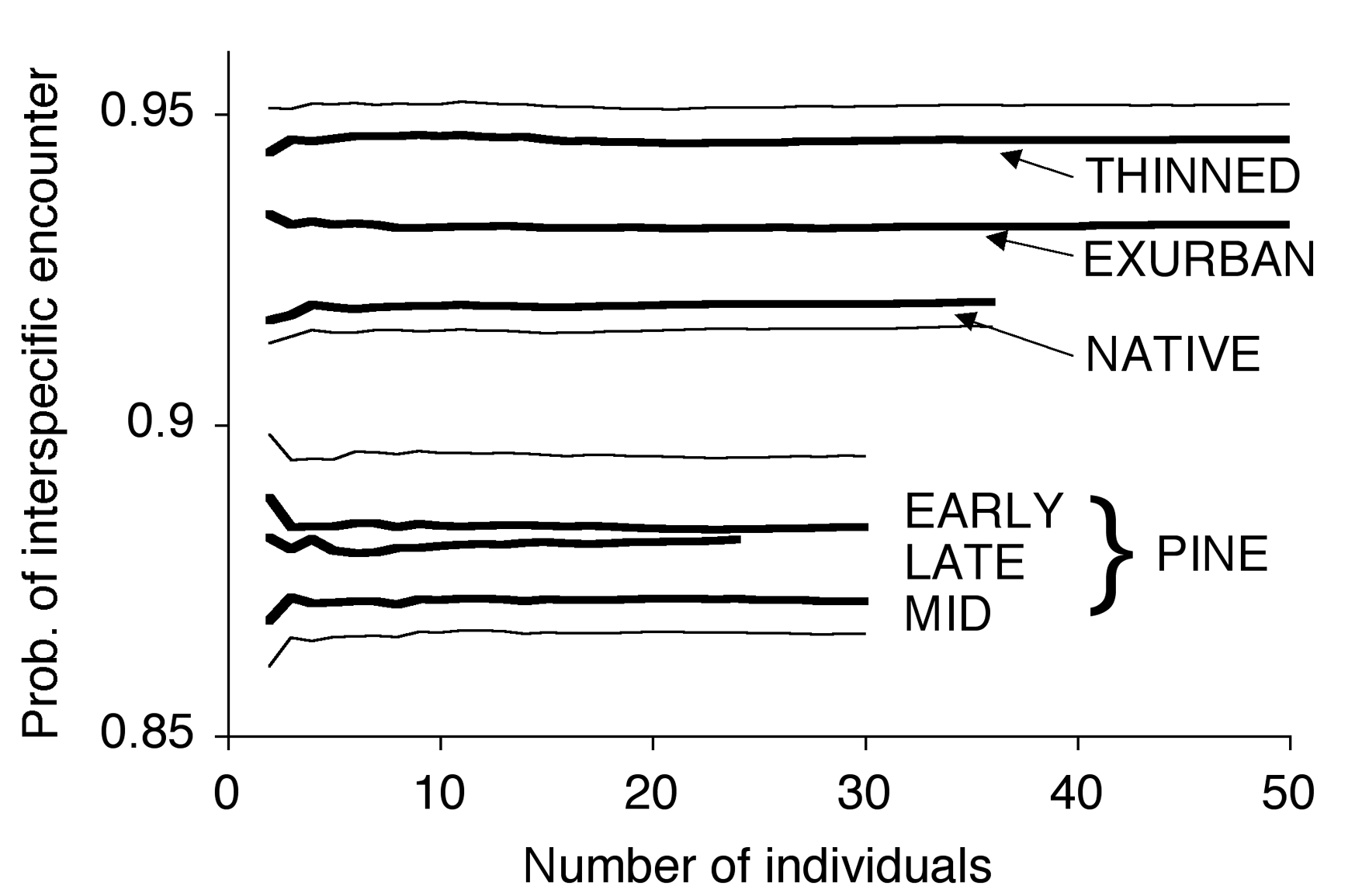

Supplement: Figure S3. Species evenness in each habitat class, calculated at the scale of transects. — Evenness is shown by the probability of interspecific encounter (PIE). Thick lines indicate means of rarefaction curves calculated for each transect and thin lines indicate 95% confidence intervals. PIE controls for both sampling effort and bird density, and uses repeated re-sampling of the data to calculate the probability that the next bird sampled will be of a different species. Therefore, high PIE values indicate high species evenness. (0.17 MB TIF) [file pone.0000063.s003.tif]

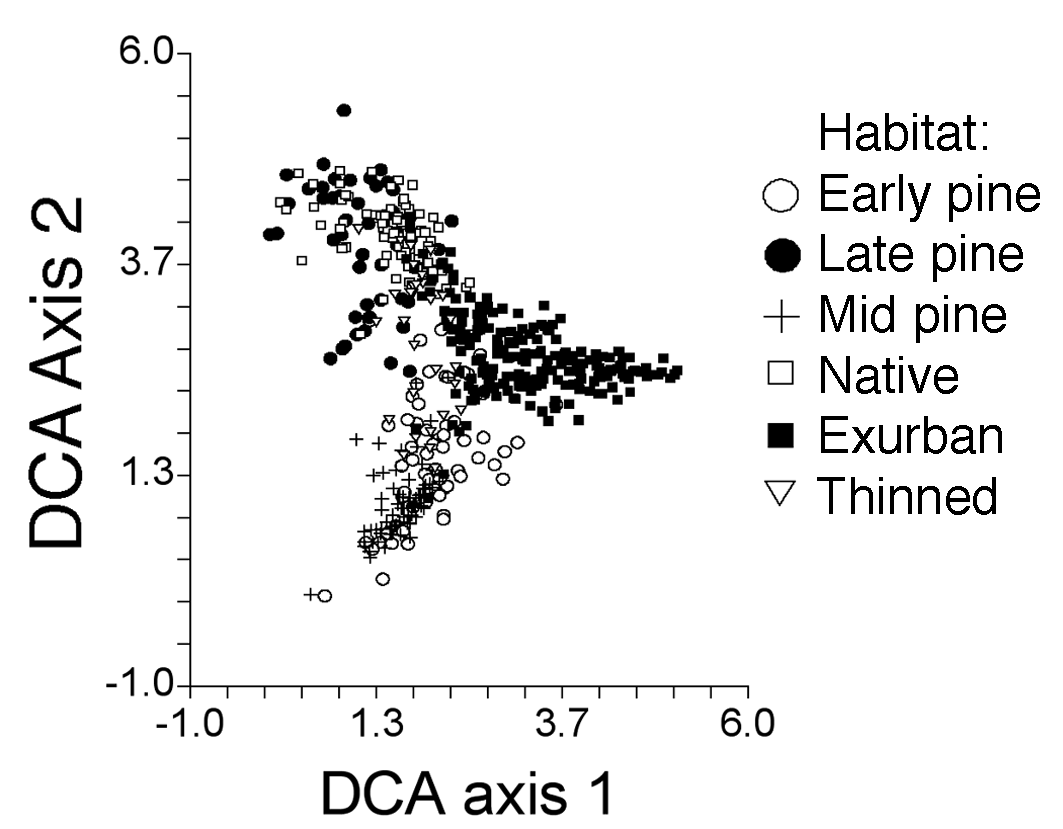

Supplement: Figure S4. Detrended correspondence analysis of bird communities calculated at the scale of individual points. — Each point represents the position in ordination space of the bird community detected at one point count. The two axes show the relative position of each point count in the multi-dimensional space defined by the species found at each point count. Thus point counts with similar bird communities cluster together on the graph. The first axis (DCA 1) is the one along which most of the variation in the ordination space is arranged (eigenvalue = 0.62), the second axis (DCA 2) is the second most important axis through the ordination space (eigenvalue = 0.52). (0.51 MB TIF) [file pone.0000063.s004.tif]
