# Supplementary material for: Depauperate Avifauna in Plantations Compared to Forests and Exurban Areas
Source: PLoS One. 2006 Dec 20;1(1):e63. doi: 10.1371/journal.pone.0000063 (PMC1762314; doi:10.1371/journal.pone.0000063)
Supplement: Table S1. Indices of abundance for each habitat class. — Two numbers are listed for each species in each habitat. The first is the proportion of points in that habitat in which the species was detected. The second is the number of birds of each species detected within 50 m per point for each habitat class divided by the number of counts and the area of the 50 m radius circle. Note that these indices are affected by differential detectability in each habitat and true density will therefore differ from these indices (see Caveats section of the Discussion for further details on detectability). * indicates species detected while traveling between point counts, but not detected during any point counts. These species were not included in statistical analyses. (0.23 MB DOC) [file pone.0000063.s005.doc]

Table S1. Indices of abundance for each habitat class.

| **Common Name** | **Scientific Name** | **Early (n = 69 sample points)** | **Late (n = 54 sample points)** | **Mid (n = 75 sample points)** | **Native (n = 85 sample points)** | **Exurban (n = 190 sample points)** | **Thinned (n = 30 sample points)** |
| --- | --- | --- | --- | --- | --- | --- | --- |
| Acadian Flycatcher | *Empidonax flaviventris* | 0.00, 0.00 | 0.00, 0.00 | 0.00, 0.00 | 0.04, 0.04 | 0.00, 0.00 | 0.00, 0.00 |
| American Crow | *Corvus brachyrhynchos* | 0.16, 0.20 | 0.19, 0.26 | 0.13, 0.15 | 0.38, 0.54 | 0.44, 0.70 | 0.43, 0.80 |
| American Goldfinch | *Carduelis tristis* | 0.16, 0.23 | 0.06, 0.06 | 0.32, 0.41 | 0.16, 0.27 | 0.19, 0.31 | 0.20, 0.30 |
| American Kestrel | *Falco sparvarius* | 0.00, 0.00 | 0.00, 0.00 | 0.00, 0.00 | 0.00, 0.00 | 0.00, 0.00 | 0.03, 0.03 |
| American Redstart | *Setophaga ruticilla* | 0.00, 0.00 | 0.00, 0.00 | 0.00, 0.00 | 0.00, 0.00* | 0.01, 0.01 | 0.00, 0.00 |
| American Robin | *Turdus migratorius* | 0.00, 0.00 | 0.00, 0.00 | 0.00, 0.00 | 0.01, 0.01 | 0.44, 0.58 | 0.03, 0.03 |
| Barn Swallow | *Hirundo rustica* | 0.00, 0.00 | 0.00, 0.00 | 0.00, 0.00 | 0.00, 0.00 | 0.14, 0.28 | 0.00, 0.00 |
| Barred Owl | *Strix varia* | 0.00, 0.00 | 0.00, 0.00 | 0.00, 0.00 | 0.01, 0.01 | 0.00, 0.00* | 0.00, 0.00 |
| Belted Kingfisher | *Ceryle alycon* | 0.00, 0.00 | 0.00, 0.00 | 0.00, 0.00 | 0.00, 0.00 | 0.01, 0.01 | 0.00, 0.00 |
| Black-and-white Warbler | *Mniotilta varia* | 0.00, 0.00 | 0.04, 0.04 | 0.01, 0.01 | 0.09, 0.11 | 0.04, 0.04 | 0.13, 0.13 |
| Black-throated Green Warbler | *Dendroica virens* | 0.00, 0.00 | 0.00, 0.00 | 0.00, 0.00 | 0.04, 0.04 | 0.00, 0.00 | 0.03, 0.03 |
| Blue Grosbeak | *Cyanocompsa parellina* | 0.00, 0.00 | 0.00, 0.00 | 0.00, 0.00* | 0.00, 0.00 | 0.02, 0.02 | 0.00, 0.00 |
| Blue Jay | *Cyanocitta cristata* | 0.01, 0.01 | 0.22, 0.26 | 0.00, 0.00 | 0.18, 0.21 | 0.18, 0.22 | 0.20, 0.33 |
| Blue-gray Gnatcatcher | *Polioptila caerulea* | 0.00, 0.00 | 0.00, 0.00 | 0.03, 0.04 | 0.05, 0.06 | 0.16, 0.18 | 0.37, 0.43 |
| Blue-headed Vireo | *Vireo solitarius* | 0.00, 0.00 | 0.02, 0.02 | 0.00, 0.00 | 0.05, 0.06 | 0.00, 0.00 | 0.00, 0.00 |
| Blue-winged Warbler | *Vermivora pinus* | 0.00, 0.00 | 0.00, 0.00 | 0.01, 0.01 | 0.00, 0.00* | 0.00, 0.00 | 0.00, 0.00 |
| Broad-winged Hawk | *Buteo platypterus* | 0.00, 0.00 | 0.00, 0.00 | 0.00, 0.00 | 0.00, 0.00 | 0.01, 0.01 | 0.03, 0.03 |
| Brown Thrasher | *Toxostoma rufum* | 0.01, 0.01 | 0.00, 0.00 | 0.00, 0.00 | 0.00, 0.00 | 0.06, 0.07 | 0.00, 0.00 |
| Brown-headed Cowbird | *Molothrus ater* | 0.01, 0.01 | 0.00, 0.00 | 0.01, 0.01 | 0.01, 0.01 | 0.16, 0.22 | 0.07, 0.07 |
| Canada Goose | *Branta canadensis* | 0.00, 0.00 | 0.00, 0.00 | 0.00, 0.00 | 0.00, 0.00 | 0.02, 0.02 | 0.00, 0.00 |
| Carolina Chickadee | *Poecile carolinensis* | 0.03, 0.04 | 0.24, 0.28 | 0.09, 0.15 | 0.22, 0.34 | 0.22, 0.29 | 0.23, 0.40 |
| Carolina Wren | *Thryothorus ludovicianus* | 0.04, 0.04 | 0.00, 0.00 | 0.04, 0.04 | 0.06, 0.06 | 0.30, 0.35 | 0.43, 0.50 |
| Cedar Waxwing | *Bombycilla cedrorum* | 0.00, 0.00 | 0.00, 0.00 | 0.00, 0.00 | 0.00, 0.00 | 0.03, 0.08 | 0.00, 0.00 |
| Chimney Swift | *Chaetura pelagica* | 0.00, 0.00 | 0.00, 0.00 | 0.01, 0.01 | 0.01, 0.01 | 0.23, 0.34 | 0.03, 0.03 |
| Chipping Sparrow | *Spizella passerina* | 0.00, 0.00 | 0.00, 0.00 | 0.00, 0.00 | 0.00, 0.00 | 0.34, 0.47 | 0.10, 0.20 |
| Common Grackle | *Quiscalus quiscula* | 0.00, 0.00 | 0.00, 0.00 | 0.00, 0.00 | 0.04, 0.05 | 0.20, 0.39 | 0.00, 0.00 |
| Common Yellowthroat | *Geothlypis trichas* | 0.28, 0.30 | 0.04, 0.06 | 0.32, 0.33 | 0.00, 0.00 | 0.26, 0.31 | 0.20, 0.20 |
| Downy Woodpecker | *Picoides pubescens* | 0.00, 0.00 | 0.00, 0.00 | 0.01, 0.01 | 0.05, 0.05 | 0.15, 0.15 | 0.17, 0.20 |
| Eastern Bluebird | *Sialia sialis* | 0.16, 0.17 | 0.00, 0.00 | 0.07, 0.07 | 0.00, 0.00 | 0.16, 0.17 | 0.00, 0.00 |
| Eastern Kingbird | *Tyrannus tryannus* | 0.03, 0.03 | 0.00, 0.00 | 0.00, 0.00 | 0.00, 0.00 | 0.05, 0.06 | 0.00, 0.00 |
| Eastern Meadowlark | *Sturnella magna* | 0.01, 0.01 | 0.00, 0.00 | 0.00, 0.00 | 0.00, 0.00 | 0.12, 0.15 | 0.00, 0.00 |
| Eastern Phoebe | *Sayornis phoebe* | 0.00, 0.00 | 0.00, 0.00 | 0.00, 0.00 | 0.01, 0.01 | 0.02, 0.03 | 0.00, 0.00 |
| Eastern Towhee | *Pipilo erthyrophthalmus* | 0.16, 0.16 | 0.04, 0.04 | 0.37, 0.47 | 0.01, 0.01 | 0.14, 0.15 | 0.40, 0.53 |
| Eastern Wood-pewee | *Contopus virens* | 0.00, 0.00 | 0.00, 0.00 | 0.00, 0.00 | 0.06, 0.06 | 0.25, 0.27 | 0.13, 0.13 |
| European Starling | *Sturnus vulgaris* | 0.00, 0.00 | 0.00, 0.00 | 0.00, 0.00 | 0.00, 0.00 | 0.39, 2.06 | 0.00, 0.00 |
| Field Sparrow | *Spizella pusilla* | 0.51, 0.58 | 0.00, 0.00 | 0.45, 0.61 | 0.00, 0.00 | 0.08, 0.09 | 0.17, 0.20 |
| Golden-winged Warbler | *Vermivora chrysoptera* | 0.00, 0.00 | 0.00, 0.00 | 0.01, 0.01 | 0.00, 0.00* | 0.00, 0.00 | 0.00, 0.00 |
| Gray Catbird | *Dumetella carolinensis* | 0.00, 0.00 | 0.00, 0.00 | 0.00, 0.00 | 0.00, 0.00 | 0.03, 0.03 | 0.00, 0.00 |
| Great Blue Heron | *Ardea herodias* | 0.00, 0.00 | 0.00, 0.00 | 0.00, 0.00 | 0.00, 0.00 | 0.01, 0.01 | 0.00, 0.00 |
| Great Crested Flycatcher | *Myiarchus crinitus* | 0.00, 0.00 | 0.00, 0.00 | 0.00, 0.00 | 0.05, 0.05 | 0.12, 0.12 | 0.07, 0.07 |
| Green Heron | *Butorides virescens* | 0.00, 0.00 | 0.00, 0.00 | 0.00, 0.00 | 0.00, 0.00 | 0.01, 0.01 | 0.00, 0.00 |
| Hairy Woodpecker | *Picoides villosus* | 0.00, 0.00 | 0.00, 0.00 | 0.00, 0.00 | 0.05, 0.05 | 0.00, 0.00 | 0.00, 0.00 |
| Hooded Warbler | *Wilsonia citrina* | 0.00, 0.00 | 0.19, 0.20 | 0.05, 0.07 | 0.46, 0.69 | 0.04, 0.04 | 0.43, 0.53 |
| House Finch | *Carpodacus mexicanus* | 0.00, 0.00 | 0.00, 0.00 | 0.00, 0.00 | 0.00, 0.00 | 0.07, 0.14 | 0.00, 0.00 |
| House Sparrow | *Passer domesticus* | 0.00, 0.00 | 0.00, 0.00 | 0.00, 0.00 | 0.00, 0.00 | 0.17, 0.37 | 0.00, 0.00 |
| House Wren | *Troglodytes aedon* | 0.00, 0.00 | 0.00, 0.00 | 0.00, 0.00 | 0.00, 0.00 | 0.01, 0.01 | 0.03, 0.03 |
| Indigo Bunting | *Passerina cyanea* | 0.70, 0.96 | 0.22, 0.24 | 0.72, 0.93 | 0.19, 0.21 | 0.60, 0.88 | 0.83, 1.60 |
| Kentucky Warbler | *Oporornis formosus* | 0.00, 0.00 | 0.06, 0.06 | 0.00, 0.00 | 0.09, 0.09 | 0.03, 0.03 | 0.03, 0.03 |
| Killdeer | *Charadrius vociferus* | 0.04, 0.09 | 0.00, 0.00 | 0.00, 0.00 | 0.00, 0.00 | 0.09, 0.10 | 0.03, 0.03 |
| Mourning Dove | *Zenaida macroura* | 0.12, 0.14 | 0.06, 0.06 | 0.27, 0.32 | 0.02, 0.02 | 0.17, 0.19 | 0.20, 0.20 |
| Northern Bobwhite | *Colinus virginianus* | 0.03, 0.03 | 0.00, 0.00 | 0.04, 0.04 | 0.00, 0.00 | 0.07, 0.09 | 0.03, 0.03 |
| Northern Cardinal | *Cardinalis cardinalis* | 0.00, 0.00 | 0.00, 0.00 | 0.03, 0.03 | 0.02, 0.02 | 0.35, 0.41 | 0.13, 0.13 |
| Northern Flicker | *Colaptes auratus* | 0.00, 0.00 | 0.00, 0.00 | 0.00, 0.00 | 0.00, 0.00 | 0.01, 0.01 | 0.00, 0.00 |
| Northern Mockingbird | *Mimus polyglottos* | 0.00, 0.00 | 0.00, 0.00 | 0.00, 0.00 | 0.00, 0.00 | 0.07, 0.09 | 0.00, 0.00 |
| Northern Parula | *Parula americana* | 0.00, 0.00 | 0.00, 0.00 | 0.00, 0.00 | 0.04, 0.04 | 0.01, 0.01 | 0.03, 0.03 |
| Orchard Oriole | *Icterus gularis* | 0.00, 0.00 | 0.00, 0.00 | 0.00, 0.00 | 0.00, 0.00 | 0.02, 0.02 | 0.00, 0.00 |
| Ovenbird | *Seiurus aurocapillus* | 0.00, 0.00 | 0.46, 0.56 | 0.00, 0.00 | 0.53, 0.74 | 0.08, 0.10 | 0.47, 0.63 |
| Pileated Woodpecker | *Dryocopus pileatus* | 0.00, 0.00 | 0.02, 0.02 | 0.00, 0.00 | 0.15, 0.16 | 0.15, 0.17 | 0.20, 0.23 |
| Pine Warbler | *Dendroica pinus* | 0.04, 0.04 | 0.48, 0.56 | 0.01, 0.01 | 0.25, 0.33 | 0.02, 0.03 | 0.40, 0.60 |
| Prairie Warbler | *Dendroica discolor* | 0.26, 0.28 | 0.07, 0.07 | 0.83, 1.40 | 0.00, 0.00 | 0.03, 0.03 | 0.33, 0.67 |
| Purple Martin | *Progne subis* | 0.00, 0.00 | 0.00, 0.00 | 0.00, 0.00 | 0.00, 0.00 | 0.04, 0.13 | 0.00, 0.00 |
| Red-bellied Woodpecker | *Melanerpes carolinus* | 0.00, 0.00 | 0.00, 0.00 | 0.00, 0.00 | 0.02, 0.02 | 0.15, 0.15 | 0.03, 0.03 |
| Red-eyed Vireo | *Vireo olivaceus* | 0.22, 0.22 | 0.57, 0.65 | 0.05, 0.05 | 0.75, 1.14 | 0.36, 0.45 | 0.80, 1.10 |
| Red-headed Woodpecker | *Melanerpes erythrocephalus* | 0.01, 0.01 | 0.00, 0.00 | 0.01, 0.01 | 0.00, 0.00* | 0.01, 0.02 | 0.00, 0.00 |
| Red-shouldered Hawk | *Buteo lineatus* | 0.00, 0.00 | 0.00, 0.00 | 0.00, 0.00 | 0.05, 0.05 | 0.03, 0.04 | 0.03, 0.03 |
| Red-winged Blackbird | *Agelaius phoeniceus* | 0.00, 0.00 | 0.00, 0.00 | 0.00, 0.00 | 0.00, 0.00 | 0.14, 0.32 | 0.00, 0.00 |
| Rock Pigeon | *Columbia livia* | 0.00, 0.00 | 0.00, 0.00 | 0.00, 0.00 | 0.00, 0.00 | 0.02, 0.03 | 0.00, 0.00 |
| Ruby-throated Hummingbird | *Archilocus colubris* | 0.00, 0.00 | 0.00, 0.00 | 0.00, 0.00 | 0.02, 0.02 | 0.01, 0.01 | 0.00, 0.00 |
| Scarlet tanager | *Piranga olivacea* | 0.03, 0.03 | 0.22, 0.24 | 0.00, 0.00 | 0.48, 0.61 | 0.06, 0.07 | 0.23, 0.30 |
| Song sparrow | *Melospiza melodia* | 0.00, 0.00 | 0.00, 0.00 | 0.00, 0.00 | 0.00, 0.00 | 0.45, 0.63 | 0.00, 0.00 |
| Summer tanager | *Piranga rubra* | 0.00, 0.00 | 0.00, 0.00 | 0.00, 0.00 | 0.04, 0.04 | 0.01, 0.01 | 0.00, 0.00 |
| Tufted Titmouse | *Baeolophus bicolor* | 0.00, 0.00 | 0.00, 0.00 | 0.00, 0.00 | 0.33, 0.40 | 0.35, 0.46 | 0.20, 0.27 |
| Turkey Vulture | *Cathartes aura* | 0.00, 0.00 | 0.00, 0.00 | 0.00, 0.00 | 0.00, 0.00 | 0.02, 0.03 | 0.00, 0.00 |
| White-breasted Nuthatch | *Sitta carolinensis* | 0.00, 0.00 | 0.00, 0.00 | 0.00, 0.00 | 0.19, 0.21 | 0.25, 0.29 | 0.23, 0.23 |
| White-eyed Vireo | *Vireo griseus* | 0.00, 0.00 | 0.00, 0.00 | 0.31, 0.33 | 0.00, 0.00 | 0.07, 0.08 | 0.20, 0.20 |
| Wild Turkey | *Meleagris gallopavo* | 0.03, 0.03 | 0.00, 0.00 | 0.00, 0.00 | 0.00, 0.00* | 0.01, 0.01 | 0.00, 0.00 |
| Wood Thrush | *Hylocichla mustelina* | 0.00, 0.00 | 0.02, 0.02 | 0.00, 0.00 | 0.15, 0.21 | 0.17, 0.18 | 0.20, 0.20 |
| Worm-eating Warbler | *Helmitheros vermivora* | 0.00, 0.00 | 0.00, 0.00 | 0.00, 0.00 | 0.06, 0.06 | 0.00, 0.00 | 0.00, 0.00 |
| Yellow-billed Cuckoo | *Coccyzus americanus* | 0.00, 0.00 | 0.00, 0.00 | 0.00, 0.00 | 0.14, 0.19 | 0.12, 0.12 | 0.23, 0.27 |
| Yellow-breasted Chat | *Icteria virens* | 0.19, 0.26 | 0.04, 0.04 | 0.73, 1.08 | 0.00, 0.00 | 0.12, 0.15 | 0.43, 0.67 |
| Yellow-throated Vireo | *Vireo flavifrons* | 0.00, 0.00 | 0.00, 0.00 | 0.00, 0.00 | 0.02, 0.02 | 0.01, 0.01 | 0.00, 0.00 |
| Yellow-throated Warbler | *Dendroica dominica* | 0.03, 0.03 | 0.00, 0.00 | 0.00, 0.00 | 0.07, 0.07 | 0.10, 0.10 | 0.53, 0.57 |

Two numbers are listed for each species in each habitat. The first is the proportion of points in that habitat in which the species was detected. The second is the number of birds of each species detected within 50 m per point for each habitat class divided by the number of counts and the area of the 50 m radius circle. Note that these indices are affected by differential detectability in each habitat and true density will therefore differ from these indices (see Caveats section of the Discussion for further details on detectability).

* indicates species detected while traveling between point counts, but not detected during any point counts. These species were not included in statistical analyses.
