# Supplementary material for: Depauperate Avifauna in Plantations Compared to Forests and Exurban Areas
Source: PLoS One. 2006 Dec 20;1(1):e63. doi: 10.1371/journal.pone.0000063 (PMC1762314; doi:10.1371/journal.pone.0000063)
Supplement: Table S2. Results of nested ANOVA on the number of species — Results are presented for birds detected within 50m of the count center and for all birds detected regardless of distance from count center. Note that these indices are affected by differential detectability in each habitat and true density will therefore differ from these indices (see Caveats section of the Discussion for further details on detectability). (0.04 MB DOC) [file pone.0000063.s006.doc]

Table S2. Results of nested ANOVA on the number of species (richness) and number of individuals (index of abundance) detected per point in six habitat classes.

| Dependent variable | Source | df | *F* | *P* |
| --- | --- | --- | --- | --- |
| Richness (50m) | Habitat class | 5 | 74.16 | <0.001 |
|  | Transect nested in habitat | 46 | 2.52 | <0.001 |
|  | Error | 451 |  |  |
| Richness (all detections) | Habitat class | 5 | 176.12 | <0.001 |
|  | Transect nested in habitat | 46 | 2.79 | <0.001 |
|  | Error | 451 |  |  |
| Index of abundance (50m) | Habitat class | 5 | 77.38 | <0.001 |
|  | Transect nested in habitat | 46 | 3.06 | <0.001 |
|  | Error | 451 |  |  |
| Index of abundance (all detections) | Habitat class | 5 | 151.31 | <0.001 |
|  | Transect nested in habitat | 46 | 3.75 | <0.001 |
|  | Error | 451 |  |  |

Results are presented for birds detected within 50m of the count center and for all birds detected regardless of distance from count center. Note that these indices are affected by differential detectability in each habitat and true density will therefore differ from these indices (see Caveats section of the Discussion for further details on detectability).
